# Supplementary figures and images for: Targeting the endolysosomal host-SARS-CoV-2 interface by clinically licensed functional inhibitors of acid sphingomyelinase (FIASMA) including the antidepressant fluoxetine
Source: Emerg Microbes Infect. 2020 Oct 17;9(1):2245–55. doi: 10.1080/22221751.2020.1829082 (PMC7594754; doi:10.1080/22221751.2020.1829082)

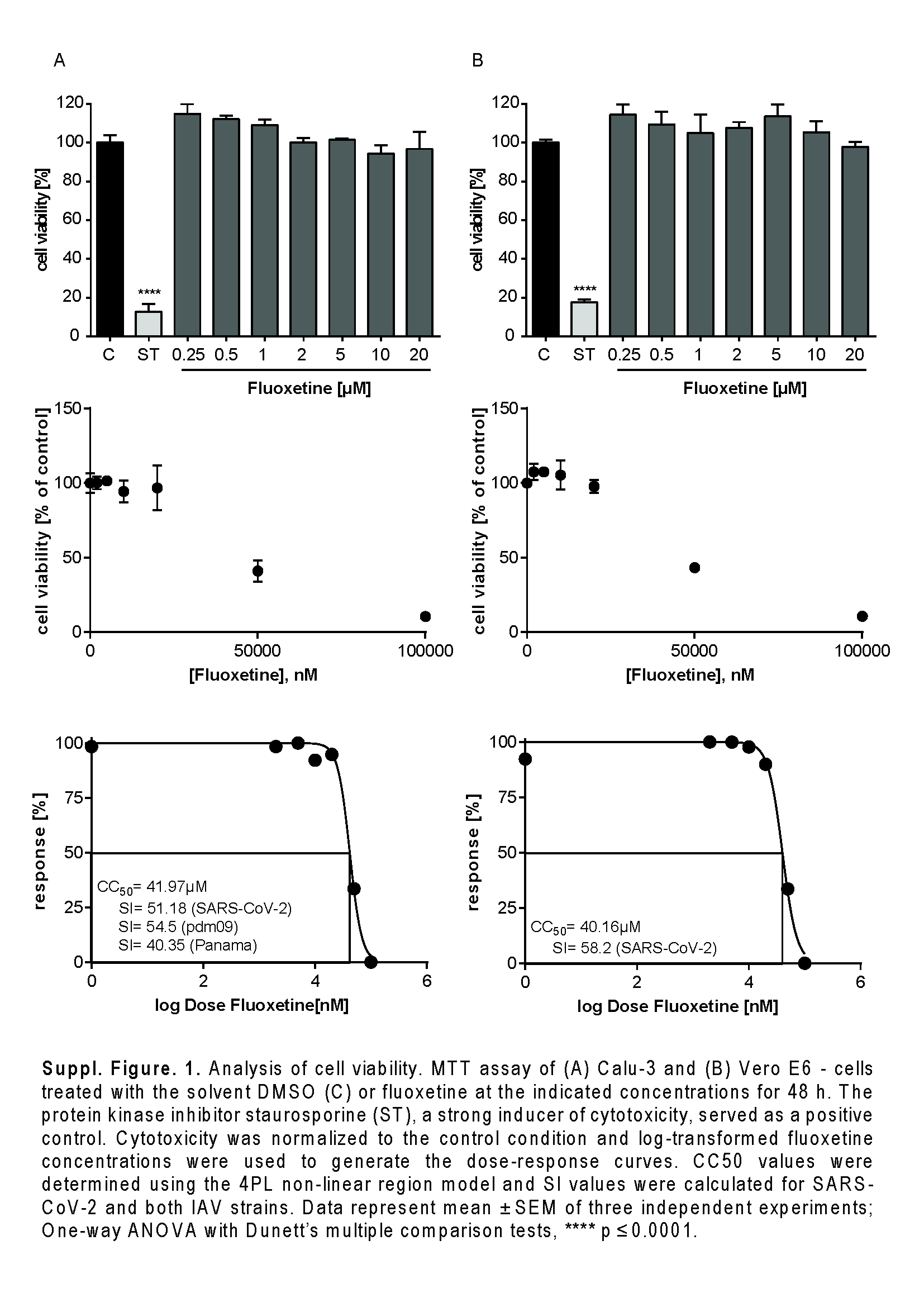

Supplement: suppl_Fig_1_final.tiff [file TEMI_A_1829082_SM4672.tiff]
